# Wax Ester Synthesis is Required for *Mycobacterium tuberculosis* to Enter *In Vitro* Dormancy

**DOI:** 10.1371/journal.pone.0051641

**Published:** 2012-12-14

**Authors:** Tatiana D. Sirakova, Chirajyoti Deb, Jaiyanth Daniel, Harminder D. Singh, Hedia Maamar, Vinod S. Dubey, Pappachan E. Kolattukudy

**Affiliations:** Burnett School of Biomedical Sciences, University of Central Florida, Orlando, Florida, United States of America; University of Delhi, India

## Abstract

*Mycobacterium tuberculosis* (*Mtb*) is known to produce wax esters (WE) when subjected to stress. However, nothing is known about the enzymes involved in biosynthesis of WE and their role in mycobacterial dormancy. We report that two putative *Mtb* fatty acyl-CoA reductase genes (*fcr*) expressed in *E. coli* display catalytic reduction of fatty acyl-CoA to fatty aldehyde and fatty alcohol. Both enzymes (FCR1/Rv3391) and FCR2/Rv1543) showed a requirement for NADPH as the reductant, a preference for oleoyl-CoA over saturated fatty acyl-CoA and were inhibited by thiol-directed reagents. We generated *Mtb* gene-knockout mutants for each reductase. Metabolic incorporation of^ 14^C-oleate into fatty alcohols and WE was severely diminished in the mutants under dormancy-inducing stress conditions that are thought to be encountered by the pathogen in the host. The fatty acyl-CoA reductase activity in cell lysates of the mutants under nitric oxide stress was significantly reduced when compared with the wild type. Complementation restored the lost activity completely in the Δ*fcr1* mutant and partially in the Δ*fcr2* mutant. WE synthesis was inhibited in both Δ*fcr* mutants. The Δ*fcr* mutants exhibited faster growth rates, an increased uptake of ^14^C-glycerol suggesting increased permeability of the cell wall, increased metabolic activity levels and impaired phenotypic antibiotic tolerance under dormancy-inducing combined multiple stress conditions. Complementation of the mutants did not restore the development of antibiotic tolerance to wild-type levels. Transcript analysis of Δ*fcr* mutants showed upregulation of genes involved in energy generation and transcription, indicating the inability of the mutants to become dormant. Our results indicate that the *fcr1* and *fcr2* gene products are involved in WE synthesis under *in vitro* dormancy-inducing conditions and that WE play a critical role in reaching a dormant state. Drugs targeted against the *Mtb* reductases may inhibit its ability to go into dormancy and therefore increase susceptibility of *Mtb* to currently used antibiotics thereby enhancing clearance of the pathogen from patients.

## Introduction

Tuberculosis is a leading cause of preventable deaths [1], [2]. The emergence of multi-drug resistant and nearly untreatable, extremely drug resistant *Mycobacterium tuberculosis* (*Mtb*) strains pose a great threat to public health [3]. The ability of the pathogen to survive in asymptomatic people in a drug-resistant, non-replicating state for decades and the inability of currently available drugs to eliminate the dormant pathogen make cure and eradication of tuberculosis extremely difficult. One third of the world population is reported to have this latent pathogen [4]. Understanding of the biochemical steps critically required for survival of the latent pathogen could help in identifying targets for novel drugs that can eliminate latent pathogen.

There is strong evidence that the pathogen uses fatty acids as the energy source for persistence in the host [5]–[9]. We showed that triacylglycerol (TAG) constitutes one storage lipid that probably serves as the source of the fatty acids [6], [10]–[12]. Wax esters (WE) constitute another form of long term storage lipid used by living organisms including bacteria [13]–[16]. WE accumulates as *Mtb* goes into a dormant state when subjected to multiple stress [11]. The biochemical processes involved in the storage and utilization of TAG in *Mtb* have been studied [6], [10]–[12], [17]. However, virtually nothing is known about the biosynthesis of WE by *Mtb* [18], even though such lipids have long been known to be present in this pathogen [19]. In other organisms in the animal and plant kingdoms, biosynthesis of WE involves acyl-CoA reduction to alcohol followed by acylation of the alcohol with fatty acyl-CoA as the substrate [20], [21]. The enzymes involved in wax synthesis and the genes that encode them in *Mtb* have not been identified.

Here we report the identification of genes which encode the fatty acyl-CoA reductases (*fcr*) that catalyze the synthesis of fatty alcohol substrates for WE biosynthesis in *Mtb*. We demonstrate that *Rv3391 (fcr1)* and *Rv1543 (fcr2)* encode acyl-CoA reductases that produce fatty alcohol from acyl-CoA. We generated *Mtb* mutant strains lacking either the Rv3391 (*fcr*1) or the Rv1543 (*fcr*2) gene and found that these mutants were severely impaired in the accumulation of fatty alcohols and WE under dormancy-inducing conditions such as combined multiple-stress (MS), nitric oxide (NO) treatment and nutrient starvation. The *fcr* deletion mutants also exhibited an increased uptake of radiolabeled glycerol and, in contrast to wild-type (WT) *Mtb*, were unable to stop multiplication under the dormancy-inducing MS condition manifesting severely diminished phenotypic antibiotic resistance development. Transcript-level measurements showed that *fcr* deficient mutants were not able to switch off transcription of genes involved in catabolism, energy generation and transcription under combined multiple-stress. Our results indicate that the *fcr1* and *fcr2* gene products are involved in the synthesis and accumulation of WE that decrease *Mtb* cell wall permeability, reduce nutrient uptake and thus inhibit replication of the pathogen.

**Figure 1:**
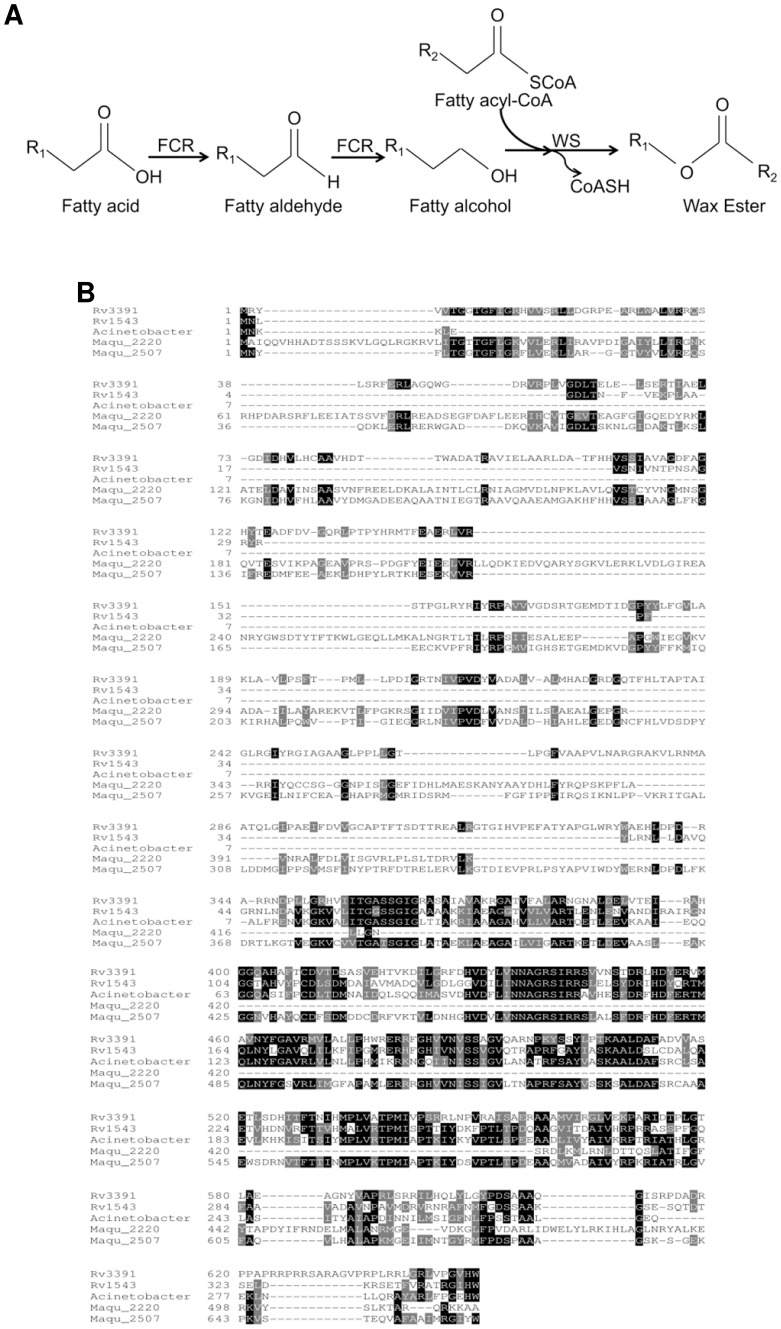
Identification of *Mtb* fatty acyl-CoA reductases. **A,** Pathway of wax ester biosynthesis. Fatty acyl-CoA reductase (FCR) catalyzes the reduction of fatty acid to fatty alcohol which is condensed with fatty acyl-CoA by wax synthase (WS) to generate wax ester. R, alkyl chain. **B,** Amino acid sequence alignment of FCR1 (Rv3391) and FCR2 (Rv1543) with the acyl-CoA reductases ACR1 of *Acinetobacter,* MAQU_2507 and MAQU_2220 of *Marinobacter*.

## Materials and Methods

### Bacterial Strains, Growth Media and Chemicals

*M. tuberculosis* H37Rv (ATCC 25618) and the *fcr* mutants were grown in Middlebrook 7H9 (Difco), Dubos–Tween–albumin medium broth (Difco) and Sauton medium, as previously described [11]. *E. coli* DH5α (Life Technologies) was used for the cloning and propagation of plasmids and phasmids. For selection of transformants, *E. coli* clones were grown in Luria–Bertani (LB) broth or on agar. When required, antibiotics were added to the culture media at the following concentrations: ampicillin: 100 µg/ml for *E. coli*, hygromycin B: 150 µg/ml for *E. coli* and 75 µg/ml for *Mtb*, and kanamycin: 50 µg/ml for *E. coli* or 20 µg/ml for *Mtb.* The NO donor (spermine NONOate) and its reference compound spermine tetrahydrochloride were purchased from Alexis Corporation. Other chemicals and antibiotics were from Sigma and Fisher Scientific. DNA restriction and modifying enzymes were obtained from New England Biolabs. Acyl-CoA substrates were purchased from American Radiolabelled Chemicals.

**Figure 2:**
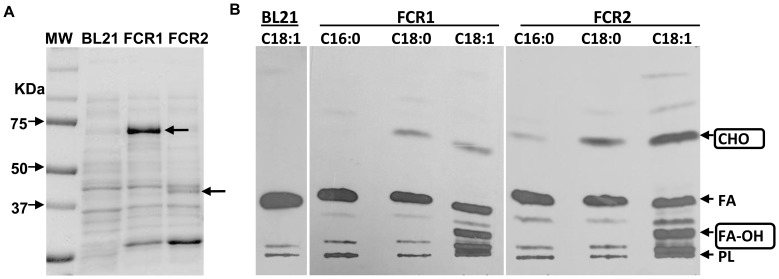
*E. coli* lysates express enzymatically active FCR1 and FCR2 proteins with negligible background activity. **A,** Cell lysates of *E. coli* expressing the reductases were analyzed by SDS-PAGE followed by coomassie staining**.** Lane 1: Molecular weight (MW) markers, Lane 2: Untransformed *E. coli* (BL21) lysate, Lane 3: FCR1-expressing *E. coli* lysate, Lane 4: FCR2-expressing *E. coli* lysate. Arrows indicate the approximately 74 kDa FCR1 protein and 40 kDa FCR2 protein. **B,** Untransformed *E. coli* BL21 lysate does not show significant acyl-CoA reductase activity. Autoradiogram of TLC analysis of reaction products shown. BL21, lysate of untransformed *E. coli* BL21 host cells; Radiolabeled palmitoyl-CoA (C16∶0), stearoyl-CoA (C18∶0) or oleoyl-CoA (C18∶1) were provided as substrates. Arrows indicate positions of authentic lipid standards. CHO, fatty aldehydes; FA, fatty acids; FA-OH, fatty alcohols; PL, polar lipids.

### Cloning and Expression of *Mtb fcr* Genes in *E.coli*

The *fcr1* and *fcr2* open reading frames (ORFs) were amplified from *Mtb* genomic DNA by PCR amplification using *PfuUltra* II fusion HS DNA polymerase (Stratagene) with the following primers (Rv3391, Forward: 5′-CACCATGCGGTACGTCGTTACCGGC-3′; Reverse:5′-CTACCAATGCACACCGGGCACCAG-3′; Rv1543, Forward:5′-CACCATGAATCTTGGT GACTTAACG-3′; Reverse: 5′-TTTTCACCAATGGATCCCTCGGGT-3′) and were cloned into pET200D-TOPO expression vector (Invitrogen, CA, USA). After sequencing, *fcr1* and *fcr2* were expressed in *E. coli* BL21 Star (DE3). Overnight cultures were diluted 1∶50 in 10 ml fresh LB medium and grown to OD_600_ 0.5–0.8, and expression of the gene was induced by the addition of isopropyl-β-D-thiogalactopyranoside (IPTG) to a final concentration of 1 mM followed by incubation at 20°C for 16 h. The induced cultures were resuspended in ice-cold 100 mM Tris.HCl pH 7.0 containing protease inhibitor cocktail (Sigma, MO) and cells were lysed by sonication using a Branson Sonifier 450 (Branson Ultrasonics Corp). Crude cell lysates were prepared freshly on the day of the assay and stored on ice.

**Figure 3:**
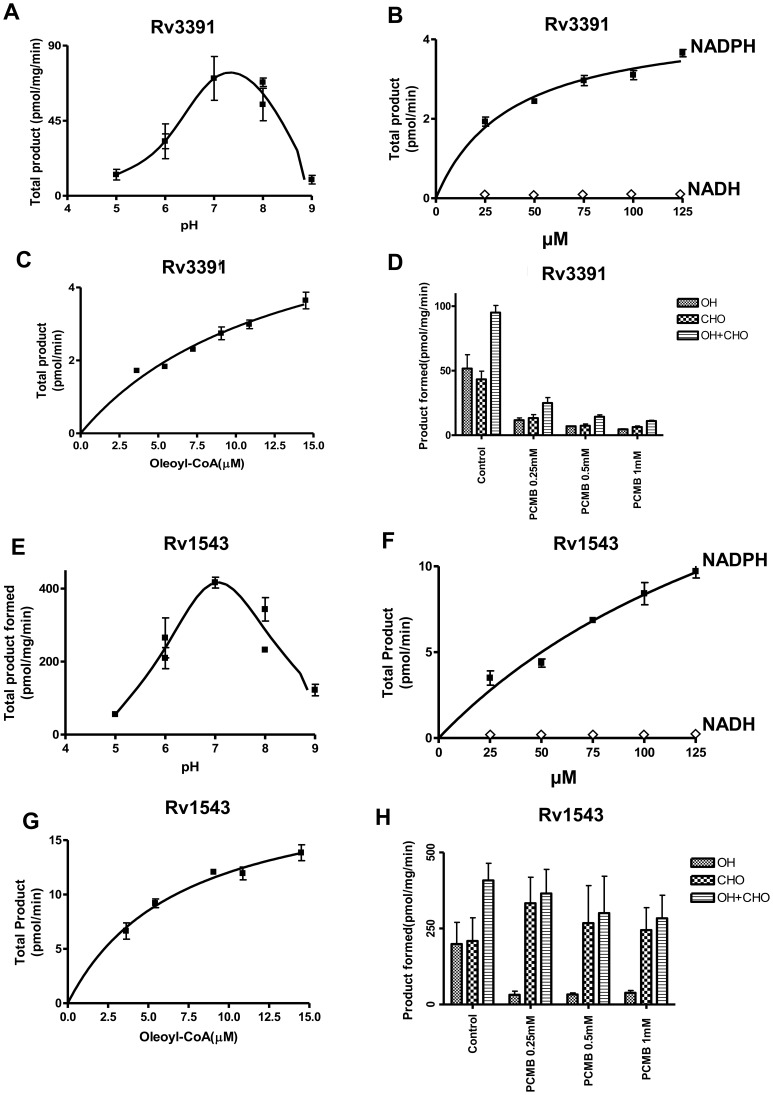
Acyl-CoA reductase activity of FCR1 and FCR2-expressing *E. coli* lysates. Acyl-CoA reductase activity was measured with 70 µg protein in a final volume of 250 µl of 0.1 M phosphate buffer for 45 min (FCR1) and 30 min (FCR2). The dependence of enzymatic activity on pH (A, E), NADPH concentration (B, F), oleoyl-CoA concentration (C, G) and inhibition of alcohol and aldehyde formation by thiol-directed reagents (D, H) was determined to confirm the authenticity of the observed acyl-CoA reductase activity in lysates. Values are average ± SD from three independent experiments.

**Figure 4:**
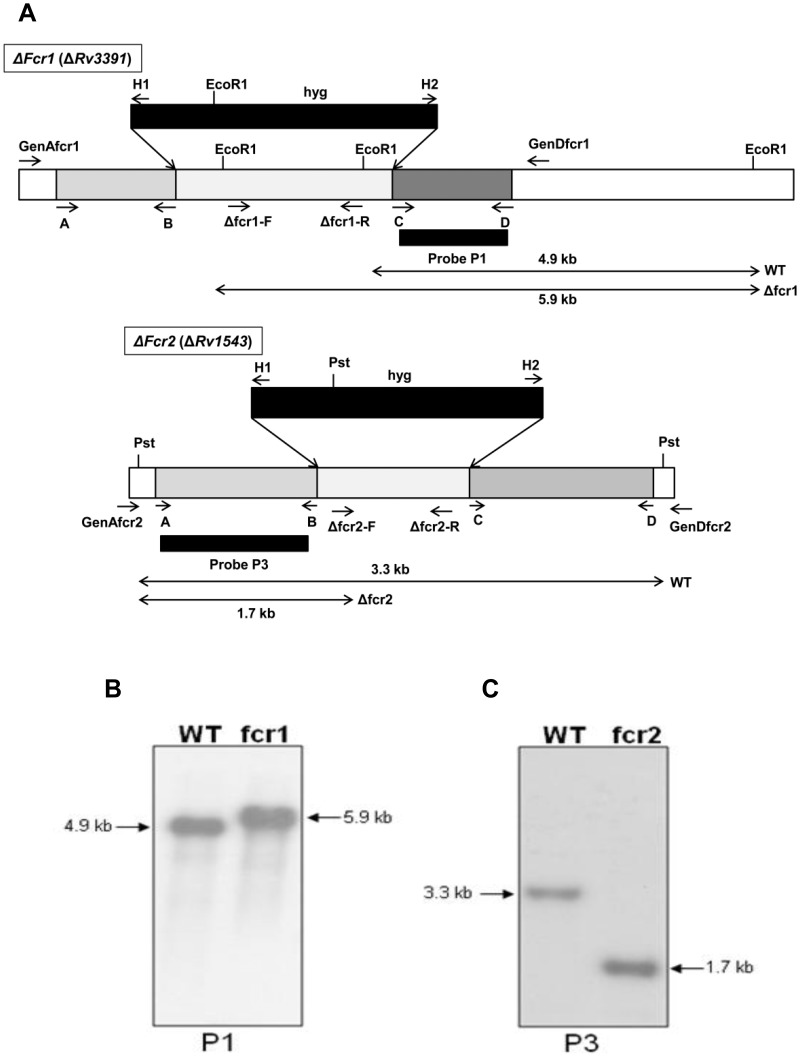
Southern blot analysis of *Mtb* Δ*fcr*1 and Δ*fcr*2 mutants. **A,** Schematic depiction shows the genomic locations of the primers and probes used in the construction and confirmation of *fcr* deletion mutants. The sequences of the primers are given in Table S1. **B,** Genomic DNA from WT *Mtb* and Δ*fcr*1 mutant was digested with *Eco*RI and hybridized with the 3′-flank of the Δ*fcr*1 construct as probe (P1). The WT *fcr1* contains two *Eco*RI sites in the deleted part of the gene, the last one being only 48 bp upstream of the 3′ flank region of the construct. When this 3′ flank sequence was used as the probe, it hybridized to a 4930 bp fragment of the *Eco*RI digested genomic DNA (lane WT). When the hyg cassette replaced the native gene sequence, its *Eco*RI site was situated 1047 bp upstream of the 3′ flank sequence which resulted in a shift of the WT band to 5929 bp (lane fcr1). **C,** Genomic DNA from WT *Mtb* and Δ*fcr*2 mutant was digested with *Pst*I and hybridized with the 5′-flank of the Δ*fcr*2 construct as the probe (P3). Wild-type genomic DNA digested with *Pst*I and probed with the 5′ flank of the disruption construct yielded a hybridization fragment of 3292 bp (lane WT). In contrast *Pst*I digested DNA from the mutant strain showed a smaller band of 1741 bp due to the presence of a *Pst*I site in the 5′ region of the hyg cassette (lane fcr2).

### Fatty Acyl-CoA Reductase Assay with *E. coli* or *Mtb* Cell Lysates

*E. coli* cultures were disrupted by sonication and *Mtb* cell lysates were prepared by disrupting the bacilli in a bead-beater blender. Lysates were maintained at ice-cold temperatures throughout lysis procedures. Lysates (50–100 µg protein) were incubated in a reaction mixture containing 100 mM Tris.HCl pH 7.0, different concentrations (0–125 µM) of NADH or NADPH and 0–15 µM [^14^C]oleoyl-CoA (55 Ci/mol; American Radiolabeled Chemicals, MO) in a total volume of 250 µl. For substrate specificity assays, [^14^C]stearoyl-CoA or [^14^C]palmitoyl-CoA (55 Ci/mol and 56 Ci/mol respectively; American Radiolabeled Chemicals, MO) were substituted in place of [^14^C]oleoyl-CoA in the above reaction mixture. After incubation for 1 h at 37°C, the reaction was terminated by the addition of 50 µl 6N HCl and lipid products were extracted with 1 ml chloroform-methanol (2∶1, by volume) followed by two extractions with 1 ml chloroform. The lipid extracts were pooled, dried under a stream of nitrogen and resolved by silica-thin layer chromatography (TLC; Analtech, DE) using hexane-diethyl ether-formic acid (40∶10:1, by volume) as a solvent system. The TLC plate was imaged by autoradiography and authentic lipid standards were used to locate the products. Radioactivity in fatty alcohol, fatty aldehyde and WE bands on TLC was determined by liquid scintillation counting. Total protein levels in respective cell lysates were determined and specific enzyme activity per mg protein was calculated to normalize the measured activity across samples. The curves were fitted using nonlinear regression analysis with the Michaelis-Menten equation (GraphPad Prism version 4; GraphPad Software, CA).

**Figure 5:**
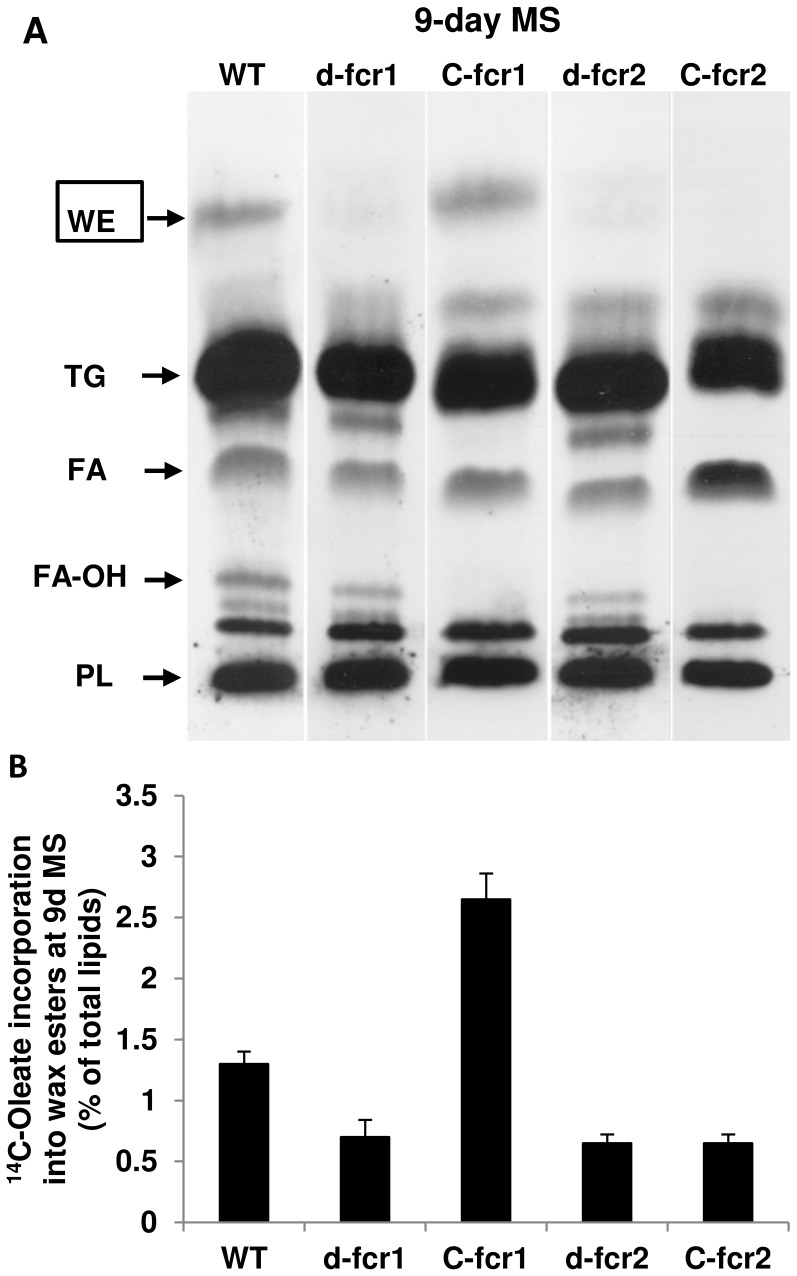
*Mtb* Δ*fcr* mutants are impaired in WE biosynthesis under combined MS. **A,** Diminished incorporation of ^14^C-oleate into WE in *Mtb* Δ*fcr* mutants under combined MS treatment. *Mtb* cultures exposed to combined MS for 9 days were metabolically labeled with ^14^C-oleic acid for 4 h. Total lipids (equal proportions across samples) were resolved on silica-TLC and autoradiograms from a typical experiment is shown. WT, wild type *Mtb*; d-fcr1, *fcr1*-deletion mutant; C-fcr1, complemented *fcr1* mutant; d-fcr2, *fcr2*-deletion mutant; C-fcr2, complemented *fcr2*-deletion mutant. Arrows indicate relative positions of authentic lipid standards. WE, wax esters; TG, triacylglycerols; FA, fatty acids; FA-OH, fatty alcohols, PL, polar lipids. **B.** Loss of *fcr1* or *fcr2* results in diminished wax ester biosynthesis under combined MS. Complementation restores WE biosynthesis in *fcr1*-deletion mutant only. Radioactivity from ^14^C-oleate incorporated into fatty alcohol and WE after 9 days under combined MS was determined and normalized as percent of total radioactivity in the respective lipid extract. Values are average ± SD.

### Preparation of [1-^14^C]oleyl Aldehyde

[1-^14^C]Oleic acid (55 Ci/mole; American Radiolabeled Chemicals) in anhydrous tetrahydrofuran was reduced with LiAlH_4_ to oleyl alcohol (55 Ci/mole) and this oleyl alcohol in CH_2_Cl_2_ was oxidized to oleyl aldehyde with pyridinium chlorochromate [22].

**Figure 6:**
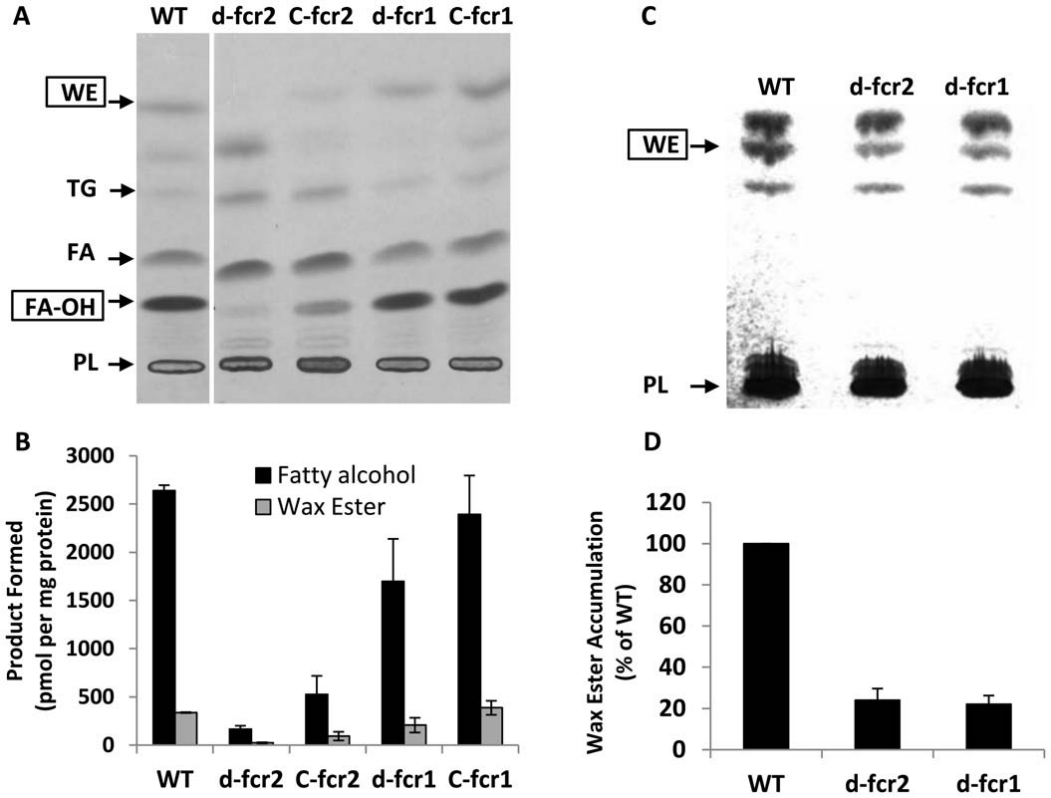
WE and fatty alcohol synthesis are impaired in *Mtb* Δ*fcr* mutants under dormancy-inducing nitric oxide treatment. **A,** Fatty alcohol and WE formation are diminished in lysates of *Mtb fcr1* and *fcr2* deletion mutants subjected to NO-stress. Complementation restores lost activity completely in *d-fcr1* and partially in *d-fcr2* lysates. *Mtb* cells were exposed to nitric oxide and lysates were assayed for acyl-CoA reductase activity using ^14^C-oleoyl-CoA as substrate as described in Methods. Autoradiogram of TLC plate from a typical experiment is shown. WT, wild-type; *d-fcr1*, *fcr1*-deletion mutant; *C-fcr1*, complemented *fcr1*-deletion mutant; *d-fcr2*, *fcr2*-deletion mutant; *C-fcr2*, complemented *fcr2*-deletion mutant. Arrows indicate relative positions of authentic lipid standards. WE, wax esters; TG, triacylglycerols; FA, fatty acids; FA-OH, fatty alcohols, PL, polar lipids. **B,** Radioactivity in WE and fatty alcohols was determined by scintillation counting and activities, normalized to total protein content in respective lysates, are shown. Values are average ± SD from duplicate experiments. **C,** NO-treated *Mtb* cells displayed severe decrease in non-radiolabeled WE accumulation. The non-radiolabeled total lipids, resolved on silica-TLC, were visualized by charring at 180°C and the TLC plate is shown. WE, wax esters; PL, polar lipids. **D,** the WE band in each lane of the TLC was quantitated by densitometry using an AlphaInnotech gel documentation system and the WE levels in the mutants, relative to WT (set at 100%), are represented. Values are average ± SD from duplicate experiments.

### Assay for Aldehyde Reductase

Aldehyde reductase activity was assayed by measuring conversion of [1-^14^C] oleyl aldehyde to oleyl alcohol. A reaction mixture containing different concentrations (0–15 µM) of [1-^14^C]oleyl aldehyde (55 Ci/mole) and 100 µM NADPH in a final volume of 250 µl of 0.1 M phosphate buffer, pH 8 was incubated at 20°C. Reaction was stopped and products were recovered and analyzed as indicated for the acyl-CoA reductase assay.

**Figure 7:**
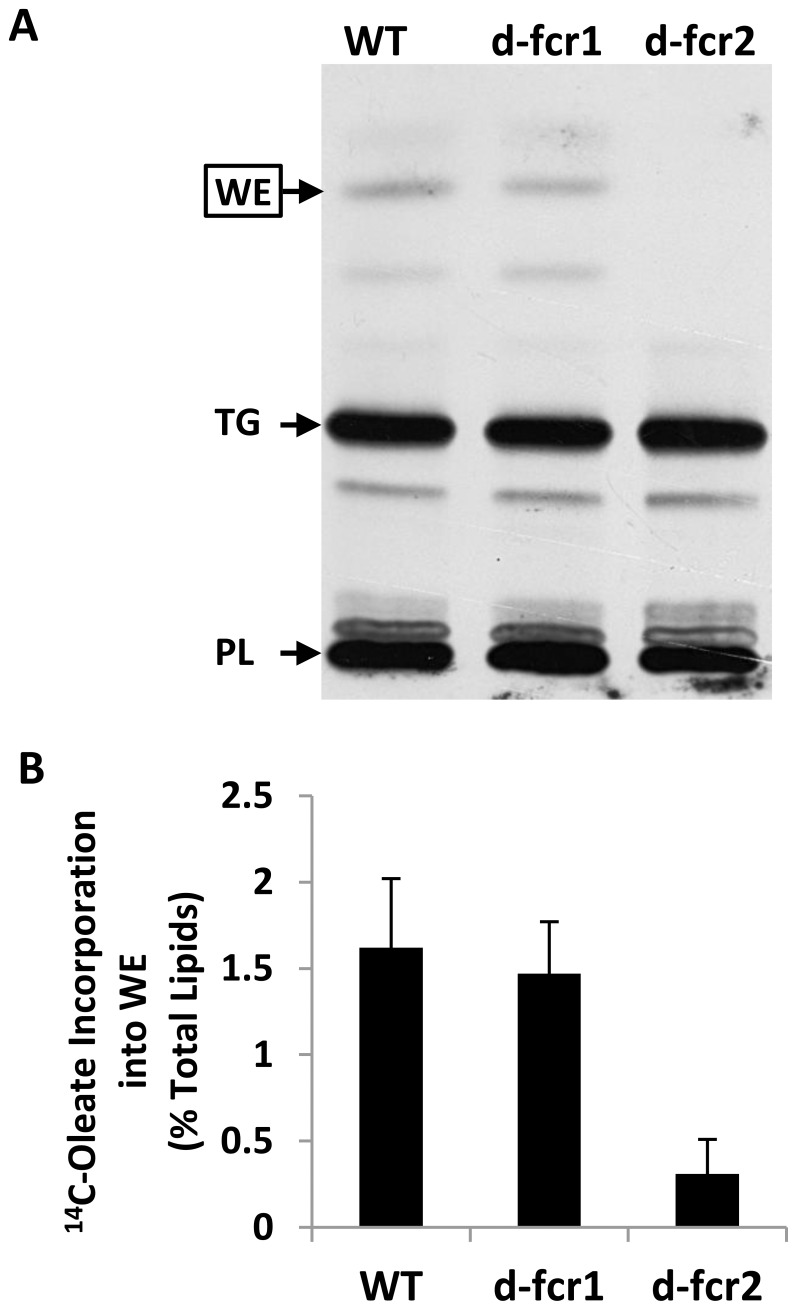
Incorporation of ^14^C-oleate into WE is severely impaired in *Mtb* Δ*fcr2* mutant starved in PBS. *Mtb* cells were grown in 7H9 medium to mid-log phase and were subjected to starvation in PBS for 72 h. **A,** Equal proportions of total lipid extracts were analyzed by TLC and a representative autoradiogram is shown. WT, wild-type; *d-fcr1*, *fcr1*-deletion mutant; *d-fcr2*, *fcr2*-deletion mutant. Arrows indicate relative positions of standard wax esters (WE), triacylglycerols (TAG) and polar lipids (PL). **B,** Radioactivity incorporated into WE was quantitated by scintillation counting and normalized as percent of radioactivity in the respective total lipid extract. Values are average ± SD from two experiments.

**Figure 8:**
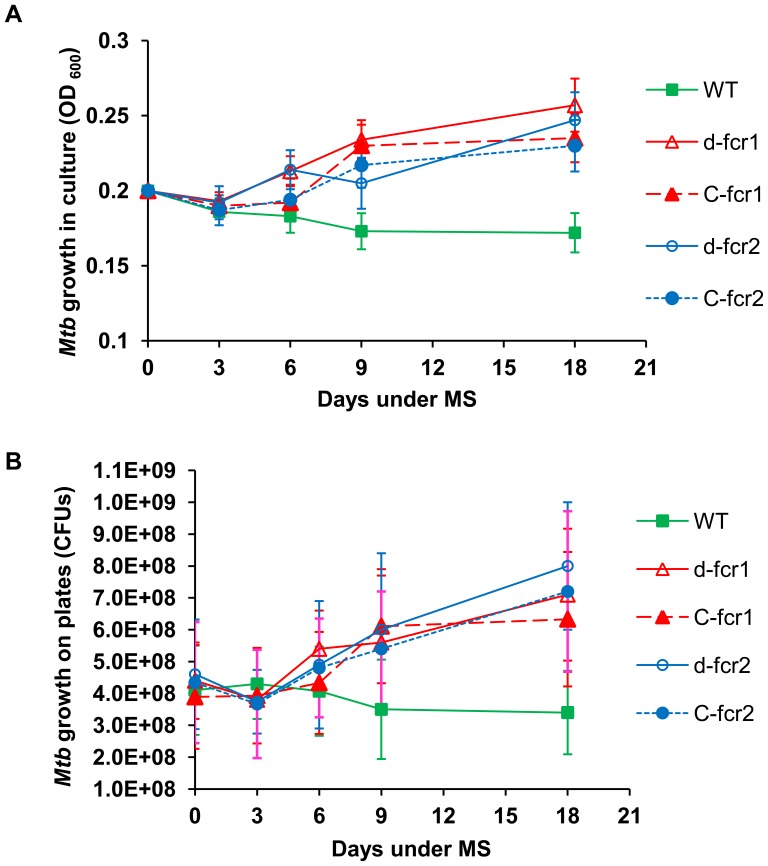
*Mtb* Δ*fcr* mutants display higher growth rates under *in vitro* dormancy. **A,** Growth (optical density at 600 nm) of *Mtb* WT, Δ*fcr* mutants and complemented mutants in liquid culture was measured at 0, 3, 6, 9 and 18 days under the MS treatment. **B,** Viable bacteria were enumerated as CFUs on 7H10-agar plates at 0, 3, 6, 9 and 18 day time points. WT, wild-type; *d-fcr1*, *fcr1*-deletion mutant; *C-fcr1*, complemented *fcr1*-deletion mutant; *d-fcr2*, *fcr2*-deletion mutant; *C-fcr2*, complemented *fcr2*-deletion mutant. Average ± standard deviation from three experiments shown (n = 3); p<0.05.

### Identification of the Aldehyde and Alcohol as Products Generated from Oleoyl-CoA

The ^14^C-labeled material that matched with co-chromatographed authentic oleyl aldehyde recovered from TLC was treated with NaBH_4_ in methanol at 20°C for one hour. The recovered reduction product was subjected to TLC along with authentic oleyl alcohol as the standard. It was then resolved using hexane:diethyl ether:formic acid (40∶10:1, v/v/v) as the solvent system. The ^14^C-labeled material that co-migrated with oleyl alcohol was treated overnight with 2∶1 v/v acetic anhydride and pyridine at 20°C. The reaction product was subjected to TLC with oleyl acetate as the authentic standard.

**Figure 9:**
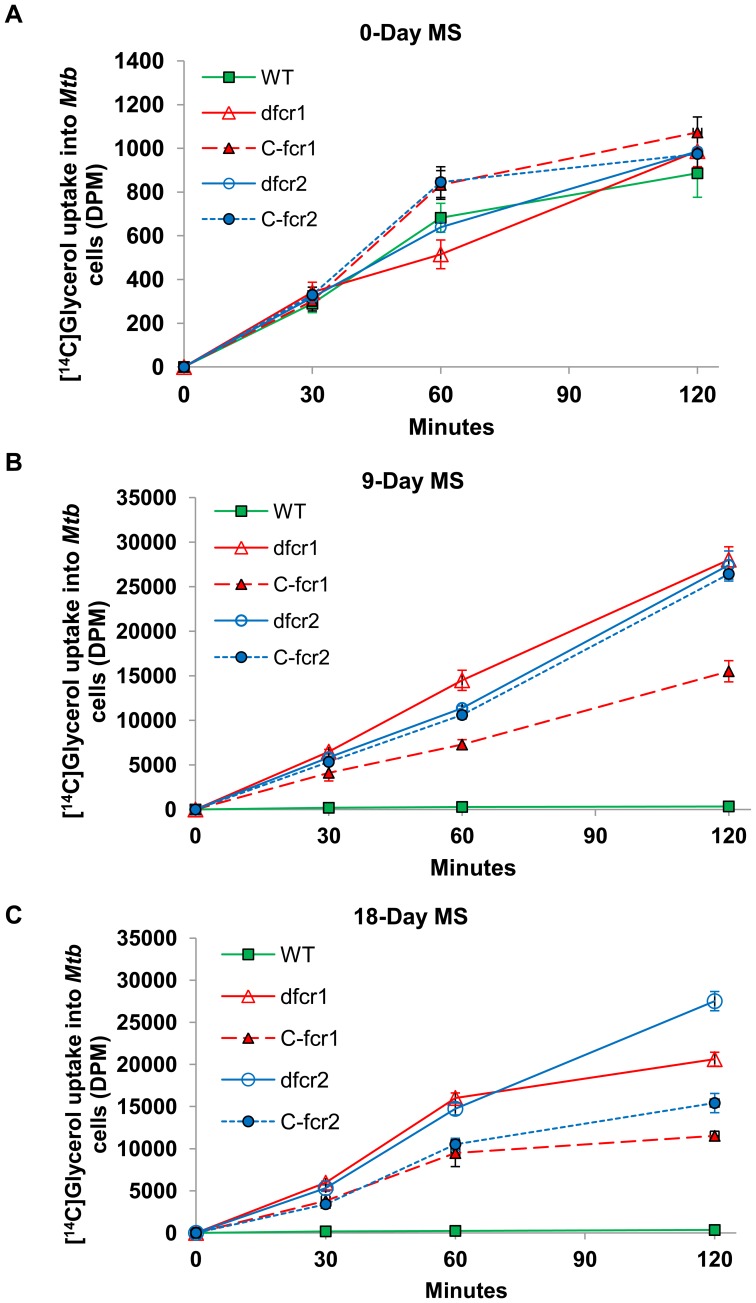
*Mtb* Δ*fcr* mutants show increased nutrient uptake under dormancy-inducing conditions *in vitro*. *Mtb* WT and *fcr*-deficient mutants and complemented mutants subjected to MS conditions were incubated with [^14^C]glycerol for different time periods. Washed cell pellets were used to measure the radioactivity inside cells as described in Methods. At 0-day, *Mtb* cells (in log-phase) displayed very low uptake of radiolabeled glycerol (**A**). At 9-days (**B**) and 18-days (**C**) under MS, in contrast to WT, *fcr*-deficient mutants and complemented mutants displayed highly elevated levels of [^14^C]glycerol uptake. WT, wild-type; *d-fcr1*, *fcr1*-deletion mutant; *C-fcr1*, complemented *fcr1*-deletion mutant; *d-fcr2*, *fcr2*-deletion mutant; *C-fcr2*, complemented *fcr2*-deletion mutant. DPM, disintegrations per minute.

### Genomic DNA Isolation and Southern Blotting

*Mtb* H37Rv genomic DNA was isolated by the guanidine thiocyanate (GTC) method using a solution of GTC, Tris/HCl and sarcosyl [23]. For Southern blot hybridization DNA samples were digested with *EcoRI* for *fcr*1 mutant and with *Pst*I for *fcr*2 mutant, subjected to 1% agarose gel electrophoresis, transferred to nylon membranes (Nytran Plus; Schleicher and Schuell), and hybridized with [α-32P] dCTP-labelled probes using the random prime labeling system Rediprime II (Amersham Pharmacia).

**Table 1:** *Mtb* Δ*fcr* mutants are severely impaired in developing phenotypic antibiotic tolerance under dormancy-inducing MS conditions.

*Mtb* strains	Days under MS	Tolerance to antibiotics (%) (± SD)	
		Rifampicin	Isoniazid
WT	9	17.0 (±5)	100
	18	25.0 (±9)	100
Δ*fcr1*	9	0.5 (±0.2)	12.0 (±4)
	18	0.8 (±0.3)	18.0 (±5)
C-*fcr1*	9	0.8 (±0.2)	25.0 (±7)
	18	1.2 (0.4)	37.0 (±9)
Δ*fcr2*	9	0.9 (±0.2)	20.0 (±6)
	18	1.4 (±0.4)	31.0 (±4)
C-*fcr2*	9	1.1 (±0.3)	28.0 (±5)
	18	1.8 (±0.4)	41.0 (±4)

*Mtb-*WT, its *fcr* gene-knockout mutants (Δ*fcr1* & Δ*fcr2*) and their complemented strains (C-*fcr1* & C-fcr2) were subjected to MS for up to 18 days and antibiotic tolerance of the cultures against rifampicin (5 µg/ml) and isoniazid (0.8 µg/ml) were measured as previously described [11]. Average (±SD) from three different experiments (n = 3) is shown. MS, multiple-stress.

### Generation of Knock-out Mutants of *Mtb*

The disrupted mutants were constructed by allelic exchange via specialized transduction using the temperature sensitive mycobacteriophage phAE159 as previously described [24]. The allelic exchange by double crossover was confirmed with two sets of primers, each representing a hygromycin (hyg) primer (primers H1 and H2) and primers in the mycobacterial genome outside the gene sequence used for making the disruption construct, GenA*fcr*1 and primers H1, H2 and GenD*fcr*2) (Table S1). The deletion mutants were selected and confirmed as previously described [24].

**Figure 10:**
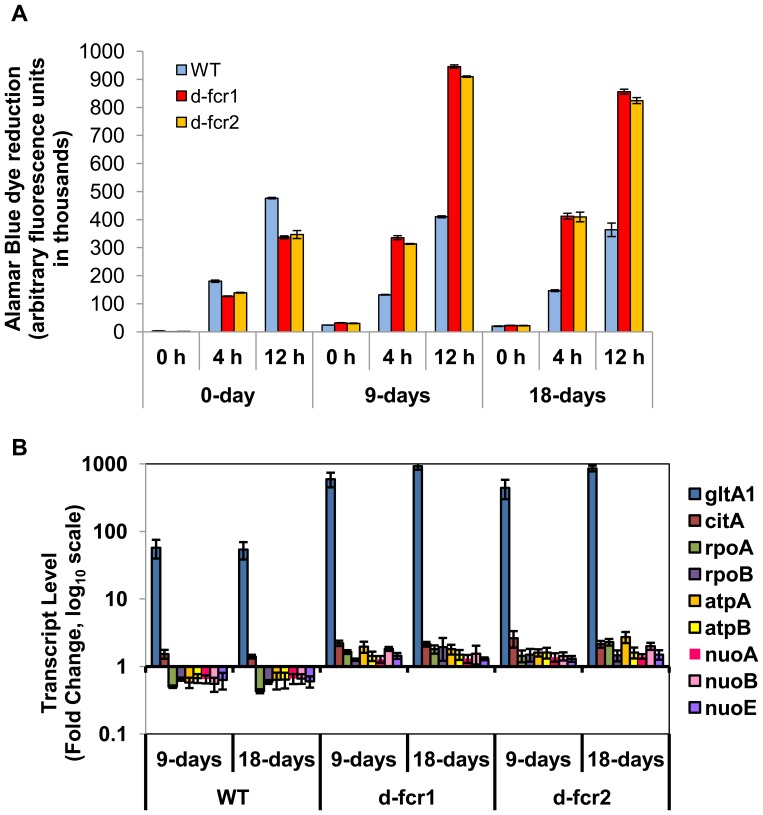
*Mtb* Δ*fcr* mutants show increased metabolic activity and induction of genes involved in energy generation and transcription under dormancy-inducing conditions. **A,** *Mtb* WT and *fcr* deficient cells subjected to combined MS condition for 0, 9 and 18 days were incubated with Alamar Blue dye for indicated periods of time and fluorescence was measured using a plate reader. **B,** Relative gene expression values (fold induction of day 0) of each gene for WT and Δ*fcr* mutants at 9 and 18 days under MS. Real-time Taqman RT-PCR measurement was performed to measure relative abundance of transcripts. Relative quantitation method (ddCt) was used with the 7900 HT real-time system and analysis was done using SDS v2.3 software of Applied Biosystems Inc. Samples of starter culture (day 0) were used as calibrator and *sigA* was used as the endogenous control to normalize expression values.

### Complementation of *fcr* Knockout Mutants

The *fcr1* and *fcr2* ORFs were cloned into the integrative mycobacterial expression vector pMV361 and the constructs were used to transform the respective *fcr* mutants. Transformants were selected on hyg-kanamycin plates and the complemented strains were cultured in the presence of both these antibiotics.

### Radio-isotopic Labeling and Lipid Analyses

*^14^C-propionate incorporation: Mtb* WT, Δ*fcr* mutants and complemented mutants were grown in 7H9 media to late log-phase (OD_600_ 1.6–1.8) and 10 ml cultures were radiolabeled with [1-^14^C]propionate (10 µCi; 55 Ci/mol; American Radiolabeled Chemicals, MO) to study their incorporation into dimycocerosyl phthiocerol. After 18 h growth in roller bottles, cells were harvested and subjected to lipid extraction in chloroform: methanol (2∶1, v/v) [25]. Total ^14^C-lipid was separated on a nonpolar solvent system using n-hexane:diethylether (9∶1, v/v) to separate dimycocerosyl phthiocerols.

*^14^C-oleate incorporation under dormancy-inducing conditions: Mtb* WT, Δ*fcr* mutants and complemented mutants were subjected to combined MS conditions, NO treatment, or starvation conditions. *Mtb* WT and *fcr* deletion mutants were subjected to combined MS as previously described [11]. Cells subjected to MS for 9 days were assayed for ^14^C-oleate incorporation into fatty alcohols and/or WE. *Mtb* WT and mutant cells (10 ml cells) were labeled with 5 µCi ^14^C-oleate (55 mCi/mmol) for 2 h. Equal quantities of *Mtb* cells (as determined by OD_600_) were used to prepare the total lipid extracts. Equal volumes of the lipid extracts were resolved by silica-thin layer chromatography (TLC) using hexane:diethyl ether:formic acid (40∶10:1, v/v/v) as solvent system. The TLC plates were autoradiographed and radioactivity in bands corresponding to authentic fatty alcohol and/or WE was determined by scintillation counting. To normalize the determined radioactive counts across samples, we expressed the radioactivity in fatty alcohol and/or WE as a fraction of the radioactivity in the respective total lipid extract loaded on a TLC lane. NO treatment was performed as previously described [12], [26]. Growth under starvation condition was done as follows: *Mtb* WT and the *fcr* mutant cells were grown in Middlebrook 7H9 to OD_600_ of 0.8, centrifuged and washed in phosphate-buffered saline (PBS) supplemented with 0.02% tyloxapol. After incubation for 72 h in PBS, 10 ml cultures were labeled with 5 µCi ^14^C-oleate and processed as described above.

For analysis of non-radiolabeled lipids, total lipids were extracted from equal quantities of *Mtb* cells (equalized by OD_600_). The dried lipid extracts were resuspended in equal volumes of chloroform-methanol (2∶1, v/v) and equal volumes were loaded on TLC plates. After resolving the lipids using hexane:diethyl ether:formic acid (40∶10:1, v/v/v) as solvent system, the TLC plates were sprayed with 5% potassium dichromate in 50% sulfuric acid and the lipids were visualized by charring at 180°C for 10 min.

### Measurement of Growth and Radiolabeled Substrate Uptake Rate under MS

To determine the changes in growth rate of *Mtb* WT, Δ*fcr* mutants and complemented mutants incubated under MS conditions, optical density and viable counts (colony forming units [CFUs] on 7H10 agar plates) were measured at different time points. Equal volumes of cultures of *Mtb* WT and Δ*fcr* mutants (adjusted to same optical density at 600 nm) from MS treated samples at different time points were incubated with 1.25 µCi of ^14^C-glycerol (130 Ci/mol; American Radiolabeled Chemicals, MO) for 30 min, 60 min and 120 min at 37°C to measure the rate of metabolic substrate incorporation. Cells were harvested and washed three times with PBS containing 0.02 percent tyloxapol, a non-ionic detergent, and total radioactivity in the washed pellets was determined.

### Assessment of Phenotypic Antibiotic Resistance Development

*Mtb* WT, Δ*fcr* mutants and complemented mutants were subjected to MS conditions for the indicated time periods and treated with rifampicin (Rif, 5 µg/ml) or isoniazid (INH, 0.8 µg/ml) for 5 additional days under the same MS as described earlier [11]. Antibiotic tolerance was determined by enumerating CFUs of the antibiotic treated and untreated (control) samples at 28 days after plating on agar plates.

### Metabolic Activity Determination

To determine the metabolic activities, the *Mtb* WT and *fcr*-deficient mutant strains were subjected to MS condition for up to 18 days. Alamar Blue dye reduction was measured using the cells after 9 and 18 day under MS and untreated cells (0 d). Cultures of different strains under MS conditions were equalized to the same optical density (OD_600_) as that of the WT and 100 µl of cell suspension was aliquoted in the 96 well plate in triplicates for each strain. To each well, 10 µl of 1∶100 diluted Alamar Blue dye suspension (Invitrogen, USA) was added, mixed and fluorescence (Excitation: 530 nm/Emission 590 nm) measured using a Bioscan Chameleon V plate reader. Fluorescence was measured at 4 h and 12 h after the addition of the dye.

### Gene Expression Analysis

*Mtb* WT, Δ*fcr* mutants and complemented mutants grown under MS condition for 0, 9 and 18 days as described above were used for RNA isolation and real-time Taqman RT-PCR analysis following our previously described protocols [11]. Real-time Taqman chemistry and 7900 HT real-time system and SDS V2.3 software of Applied Biosystems, Life Tech, USA, were used to measure relative amount of transcript levels as previously described [6].

## Results

### Identification of Putative Acyl-CoA Reductase Genes in *Mtb* Genome

Acyl-CoA reductase generates fatty alcohol from fatty acyl-CoA for the synthesis of WE (Fig. 1A). Analysis of *Mtb* genome for fatty acyl CoA reductase genes (*fcr)* revealed two ORFs encoding putative *fcr* genes [11]. The first, *fcr1* (*Rv3391, acrA1; NCBI Gene ID: 887950*) gene product is a putative protein of 650 amino acids annotated as a multifunctional enzyme with putative acyl-CoA reductase activity in the C-terminal part [27]. The N-terminal part has homology to proteins with acyl carrier protein and keto reductase domains. The second, *fcr2* (*Rv1543; NCBI Gene ID: 886400*), also annotated as a possible fatty acyl-CoA reductase encodes a 341 amino acid gene product [27]. Alignment of the two *fcr* genes showed a 39.8% identity and 71.8% similarity between *fcr2* and the C-terminal 319 amino acids of *fcr1. In silico* analysis using the TMpred Server (http://www.ch.embnet.org/software/TMPRED) indicated that FCR1 protein contained 6 putative transmembrane helices while FCR2 protein contained at least two transmembrane helices. Both *fcr* genes are conserved in *Mycobacterium leprae* and other mycobacterial species. Acyl-CoA reductases from *Marinobacter* have been shown to reduce fatty acyl-CoA to fatty alcohol without releasing fatty aldehyde intermediate whereas the *Acinetobacter* enzyme catalyzed aldehyde formation only [14], [28], [29]. The *Mtb* FCR1 shows 40% identity with the *Marinobacter* reductase (MAQU_2507; over a 639 amino acid stretch) and 41% identity with the *Acinetobacter* reductase (ACR1; over a 299 amino acid stretch). FCR2 shows 48% identity with ACR1 (over a 291 amino acid stretch) and 50% identity with Maqu_2507 (over a 310 amino acid stretch). Multiple sequence alignment of FCR1 and FCR2 with the acyl-CoA reductases ACR1 (NCBI Gene ID: 6000427) of *Acinetobacter baumannii*, MAQU_2507 (NCBI Gene ID: 4655706) and MAQU_2220 (NCBI Gene ID: 4657301) of *Marinobacter aquaeolei* VT8 is shown in Fig. 1B.

### Expression of *Mtb* FCR Proteins in *E. coli*

In other organisms, acyl-CoA reductases have been found to catalyze the reduction of fatty acyl-CoA to fatty alcohol [30], [31]. We examined whether these previously uncharacterized *Mtb* gene products, with amino acid sequence similarities to other acyl-CoA reductases, displayed such activity. Expression of *fcr1* and *fcr2* gene products in *E. coli* was high at 37°C but enzymatic activity in cell lysates was higher when expression was done at 20°C. Therefore, the ORFs were expressed at 20°C for examination of their catalytic activities. Induction of the *fcr* genes overnight followed by SDS-PAGE analysis of cell lysates showed accumulation of proteins of the expected size from the ORFs: 74 kDa for *fcr1* and 40 kDa for *fcr2* (Fig. 2A). Densitometric analysis of the lysates revealed that FCR1 accounted for nearly 39% of the total proteins and FCR2 accounted for approximately 14% of the total proteins in the respective *E. coli* BL21 lysates.

### The *Mtb* FCRs Expressed in *E. coli* Display Acyl-CoA reductase Activities

Lysates of *E. coli* BL21 cells expressing FCR1 or FCR2 showed acyl-CoA reductase activity and produced fatty aldehyde and fatty alcohol whereas the lysates of untransformed *E. coli* BL21 did not show detectable fatty acyl-CoA reductase activity (absence of fatty alcohol and aldehyde formation from [^14^C]oleoyl-CoA - Fig. 2B; lane BL21). Since the endogenous acyl-CoA reductase activity in the *E. coli* BL21 host cell lysate was negligible, we used the lysates of *E. coli* expressing FCR1 and FCR2 for the determination of appropriate assay conditions for the enzymatic activities of the expressed proteins.

Both FCR1 and FCR2 overexpressed in *E. coli* lysates preferred oleoyl-CoA as substrate over stearoyl-CoA or palmitoyl-CoA (Fig. 2B). It was found that both reductases produced alcohol and aldehyde which co-migrated with authentic oleyl alcohol (R_f_ 0.26) and oleyl aldehyde (R_f_ 0.71) respectively. The alcohol product when treated with acetic anhydride gave a product that co-migrated with authentic oleyl acetate with an R_f_ of 0.6. The product that co-migrated with oleyl aldehyde, when treated at 20°C with NaBH_4_, was converted to a product that co-migrated with authentic oleyl alcohol (data not shown). These results supported the identification of the products generated by the expressed reductases.

Both reductases showed maximum activity at about pH 7 (Figs. 3A, E). Both alcohol and aldehyde formation showed the same optimal pH. Alcohol formation by both enzymes increased rectilinearly with increasing time of incubation. Formation of the aldehyde intermediate increased during the early time periods and then gradually decreased as it was being converted to alcohol (data not shown). With both reductases, product formation taken as the sum of aldehyde and alcohol increased rectilinearly with increasing protein concentration up to 320 µg/ml. Both FCR1 and FCR2 exhibited strong preference for NADPH as a cofactor, whereas NADH was found to be ineffective (Fig. 3B, F). Both of these proteins showed typical substrate saturation with oleoyl-CoA (Fig. 3C, G). Both acyl-CoA reductases were found to be sensitive to thiol-directed reagents. *p*-Chloromercuribenzoate (PCMB) severely inhibited both alcohol and aldehyde production catalyzed by FCR1 (Fig. 3D). On the other hand, PCMB drastically decreased alcohol production catalyzed by the FCR2 but aldehyde production was not affected. (Fig. 3H).

### Generation of *fcr* Gene-disrupted Mutants

To assess the role of the *fcr* gene products in WE production in *Mtb* under stress, we generated *fcr* gene-knockout mutants by allelic exchange via specialized transduction using conditionally replicating mycobacteriophage phAE159 [32]. To prepare the *fcr1* disruption construct, a 1792 bp fragment, out of the total 1953 bp *fcr*1 ORF, was replaced with the res-hyg-res cassette in pYUB854 and used as the substrate for allelic exchange by a double crossover event by homologous recombination (Fig. 4A). Screening of the hyg-resistant transductants with a set of primers (Δ*fcr*1-F and Δ*fcr*1-R, in Table S1) specific for the deleted segment identified several mutants that failed to amplify the expected 889 bp fragment (data not shown). Disruption by homologous recombination was confirmed by further PCR analysis of the flanking regions (primer pairs GenA/H1 and H2/GenD), which yielded the expected-size products. Southern blot analysis of *Mtb* WT and the *fcr*1 mutant is shown in Fig. 4B. Similarly, 838 nucleotides sequence from the middle portion of *fcr*2 ORF was deleted and replaced by the hyg cassette. This disrupted copy of *fcr*2 was introduced into *Mtb* via the transducing mycobacteriophage phAE159 and replaced the native copy of the gene (Fig. 4C). Mutants were screened by PCR for loss of the deleted portion of the gene (700 bp, data not shown) using primers Δ*fcr*2-F and Δ*fcr*2-R (Table S1). Southern blot analysis confirmed that the mutant clone contained a single disrupted copy of the gene. The *fcr* mutants did not show any alteration in dimycoceryl phthiocerol synthesis from [1-^14^C]propionate labeling experiments (data not shown).

### The *Mtb* Δ*fcr* Mutants Show Decrease in Incorporation of ^14^C-oleate into WE, Decreased acyl-CoA Reductase Activity and Ability to Accumulate WE

We have found that under stress conditions, thought to induce dormancy, *Mtb* accumulates both TAG and WE as energy reserves [6], [10]–[12]. To test whether the *fcr* genes we identified are involved in the synthesis of fatty alcohols used for WE biosynthesis under such stress conditions, we subjected *Mtb* WT and Δ*fcr* mutants to various stresses and determined changes in fatty alcohol and/or WE synthesis. *Mtb* WT, mutants lacking *fcr1* or *fcr2* and complemented mutants were subjected to combined MS conditions designed to mimic conditions encountered by the pathogen inside the granuloma [11]. Incorporation of ^14^C-oleate into WE by the Δ*fcr1* and Δ*fcr2* mutants was significantly reduced at 9 days under combined MS (Fig. 5). Complementation restored WE biosynthesis in *fcr1*-deletion mutant only.

*Mtb* cells were subjected to nitric oxide stress that was reported to mimic the situation inside human macrophages [26]. We assayed cell lysates of *Mtb* WT, Δ*fcr* mutants and complemented mutants for radiolabeled fatty alcohol and wax ester formation from ^14^C-oleoyl-CoA. The Δ*fcr1* mutant showed a moderate decrease in fatty acyl-CoA reductase activity while the lysates from the Δ*fcr2* mutant showed a more severe loss of acyl-CoA reductase activity. Complementation of the disrupted genes with an extra-chromosomal copy restored the lost *fcr1* activity completely but the lost *fcr2* activity was only restored partially (Fig. 6A, B). Gene transcript analysis showed that disruption of *fcr1* or *fcr2* did not cause compensatory upregulation of the undisrupted paralog under MS conditions (data not shown). We analyzed lipid extracts from *Mtb* WT and Δ*fcr* mutants subjected to NO stress by silica-TLC followed by dichromate-sulfuric acid charring to visualize the lipids. In the mutants lacking either *fcr* gene, WE accumulation, as determined by TLC, was much lower when compared to the WT *Mtb* (Fig. 6C, D).

Since starvation is thought to be one of the main dormancy-inducing stresses encountered by *Mtb* inside the granuloma [11], [33], *Mtb* WT and Δ*fcr* mutants were starved in PBS to determine the role of the *fcr* genes in wax synthesis under this dormancy-inducing stress. In the Δ*fcr*1 mutant, the incorporation of^ 14^C-oleic acid into WE was only slightly lower than that observed with the WT but Δ*fcr*2 cells showed a drastic decrease in incorporation of the radiolabel into WE, compared to the WT (Fig. 7A, B).

### *Mtb* Δ*fcr* Mutants Display Continued Growth, Increased Uptake of Glycerol and Loss of Ability to Develop Phenotypic Antibiotic Resistance under Combined MS Condition

One of the important characteristic features of *Mtb* dormancy is the non-replicating state of *Mtb* cells. Under our combined MS model, the *Mtb* WT strain was able to control its replication and did not show any increase in growth measured by culture density changes and by the viable counts enumerated by counting CFUs on agar plates. In contrast, the Δ*fcr* mutants did not manifest a non-replicating state upon exposure to combined MS. They showed an increased growth rate by optical density measurements and colony counts on plates. Complementation did not restore the WT phenotype in the mutants (Fig. 8).

We postulate that WE serve as a barrier inhibiting the flow of nutrients across the *Mtb* cell wall. Deletion of *fcr1* or *fcr2* genes, which results in loss of WE synthesis, may reduce this barrier and increase nutrient flow across the *Mtb* cell wall. In support of this hypothesis, the Δ*fcr* mutants showed a much higher rate of uptake of radiolabeled glycerol as compared to the WT strain. When the WT and mutants at 9 days and 18 days of exposure to multiple stress were incubated with ^14^C-glycerol for increasing time periods, the uptake by the mutants was 30-fold higher than that of WT at 30 min and about 60-fold to 80-fold higher at 2 h (Fig. 9B, C). WT and Δ*fcr* mutants, used for MS, showed very little uptake of radiolabeled glycerol prior to exposure to MS conditions (Fig. 9A). The complemented mutants did not show restoration of the WT phenotype.

The *fcr* deletion mutants which grow more rapidly than the WT may be expected to show decreased phenotypic antibiotic tolerance. In support of our hypothesis, we observed that deletion of *fcr1* or *fcr2* resulted in severe impairment in phenotypic antibiotic resistance development under MS. Under combined MS condition, approximately 17 and 25 percent of the WT *Mtb* cells developed rifampicin tolerance at 9 and 18 days, respectively, compared to the untreated control. Under the same conditions, the entire WT *Mtb* population (100%) developed isoniazid tolerance (Table 1). In contrast, rifampicin tolerance in Δ*fcr1* mutant decreased drastically to 0.5% at 9 days and 0.8% at 18 days. Likewise, phenotypic tolerance to isoniazid was severely decreased in the Δ*fcr1* mutant. The Δ*fcr2* mutant also showed similar steep losses in phenotypic tolerance to rifampicin and isoniazid like the Δ*fcr1* mutant. The complemented mutants remained sensitive to the antibiotics (Table 1).

### Upregulation of Metabolic Activity and Induction of Genes Associated with Energy Generation and Transcription in Δ*fcr* Mutants under Dormancy-inducing Conditions

If the uptake of nutrients, such as glycerol, is increased in the *fcr* mutants, under MS conditions, then metabolic activity levels of the *fcr* mutants may be expected to be higher than *Mtb* WT. We used the Alamar Blue dye to determine metabolic activity levels of *Mtb* WT and *fcr* mutants under MS conditions. We found that the Δ*fcr* mutants showed more than 2-fold induction of metabolic activity levels compared to WT, as measured by the reduction of Alamar Blue dye by the *Mtb* cells (Fig. 10A).

To determine whether the observed hyper-growth phenotype of the Δ*fcr* mutants, that are unable to go into a dormant state under multiple-stress application, is reflected in gene expression changes, we examined the transcript levels of some genes that are mainly involved in catabolism, energy generation and transcription based on our previous study and other reports [11], [33]. Transcription levels of the following genes were elevated in the Δ*fcr* mutants in comparison to *Mtb* WT under MS conditions (Fig.10B): *gltA1* (citrate synthase I of TCA cycle; Rv1131), *citA* (citrate synthase II; Rv0889c), *rpoA* and *rpoB* (RNA polymerase genes; Rv3457c and Rv0667, respectively), *atpA* and *atpB* (ATP synthase genes; Rv1308, Rv1304 respectively), *nuoA, nuoB* and *nuoE* (NADH dehydrogenase I subunit genes; Rv3145, Rv3146 and Rv3149 respectively).

## Discussion

*Mtb* is known to accumulate WE under stress conditions that are thought to be encountered in the host by the pathogen [11]. Nothing is known about how WE are generated in *Mtb*. Biosynthesis of WE in other organisms involves three different enzymatic steps: acyl-CoA is reduced to fatty aldehyde that undergoes reduction to fatty alcohol followed by esterification by a fatty alcohol : acyl-CoA transacylation [14], [20], [21], [34], [35]. These reactions, first demonstrated in cell free extracts of *Euglena gracilis* [30] have been demonstrated in numerous organisms in both plant and animal kingdoms [14], [21], [36]–[38].

Fatty acyl-CoA can be reduced to alcohol without any significant release of fatty aldehyde intermediate as seen in *E. gracilis*, jojoba seeds and sebaceous glands [21], [30], [39]. In such cases a single protein catalyzes both acyl-CoA reduction to aldehyde and reduction of the aldehyde to alcohol [21], [30]. In other cases two separate proteins generate fatty alcohol from fatty acyl-CoA; the first one generates the aldehyde and the second catalyzes aldehyde reduction [40]. In the *Mtb* genome, *fcr1*, that would encode a 74 kDa protein, has been annotated as acyl-CoA reductase (*acrA1*) [41]. We found by homology that *fcr2* might also encode a smaller 40 kDa acyl-CoA reductase. Expression of these genes in *E. coli* and examination of their enzymatic activities show that *fcr1* and *fcr2* encode acyl-CoA reductases generating free aldehyde and fatty alcohol. Since the untransformed *E. coli* host cells showed negligible fatty acyl-CoA reductase activity, lysates of *E. coli* BL21 expressing FCR1 and FCR2 were used to establish that the two gene products behaved like acyl-CoA reductases from other organisms.

Our results showed that disruption of the reductase genes resulted in significant decreases in the synthesis of fatty alcohol and WE by *Mtb* exposed to nitric oxide, nutrient starvation or combined MS that are thought to mimic *in vivo* conditions that lead to dormancy [11], [26], [33]. Since we did not observe alteration in dimycoceryl phthiocerol synthesis from [1-^14^C]propionate labeling experiments, it is likely that FCR1 and FCR2 do not contribute to the synthesis of phthiocerol. A meta-analysis of the microarray data of *Mtb* genes under *in vitro* and *in vivo* conditions mimicking dormancy reported that *fcr1* showed a higher upregulation score than *fcr2* thereby suggesting its greater importance during dormancy [42]. Nevertheless, our data shows that *fcr2* contributes to fatty alcohol biosynthesis to a greater extent than *fcr1* under the stress conditions we tested.

The newly identified *Mtb fcr* gene products contribute to biosynthesis of WE that may serve as an additional carbon source during dormancy. When *Mtb* was subjected to *in vitro* hypoxia that was reported to induce a state of non-replicating persistence, we found that it accumulated TAG as the predominant storage lipid and that the stored TAG was utilized as an energy source during subsequent starvation [10], [17]. We also reported that, under the *in vitro* dormancy-inducing MS conditions designed to mimic the microenvironment within the human lung granuloma, the inability of *Mtb* Δ*tgs1* mutant to accumulate TAG was accompanied by a loss of antibiotic tolerance [11]. On the other hand, both WE and TAG accumulated in *Mtb* exposed to combined MS [11]. We reported recently that *Mtb* inside foamy, lipid-loaded macrophages accumulated TAG primarily [6]. WE is also synthesized, but to a minor extent. Since *Mtb* enters a dormant state inside lipid-loaded macrophages, TAG may be the major storage lipid in *Mtb* for use as an energy source during dormancy. WE are known to be one form of energy storage in both the plant and animal kingdoms [21], [37]–[39]. During germination of jojoba seeds, a WE hydrolase capable of hydrolyzing stored WE was reported to be induced [43]. Although more refractory to hydrolysis, WE could be hydrolyzed by *Mtb* lipases that hydrolyze TAG. We tested 24 *Mtb* lipases expressed in *E. coli* for their ability to hydrolyze WE and found that among them only LIPY, which is the major TAG lipase of *Mtb* [17] exhibited weak WE hydrolase activity (our unpublished observations).

WE can have other biological functions apart from acting as an energy reserve. They serve as a permeability barrier to protect organisms from desiccation by coating their surfaces [20], [37], [44]. Evaporation of moisture from tear film in the eye is retarded by the secretion of wax from the meibomian gland [21]. *Mtb* has been reported to display a marked thickening of the outer lipid membrane in a low-oxygen environment, that mimics the microenvironment within the granuloma, in which it goes into dormancy [45]. Multidrug-resistant and extremely drug-resistant *Mtb* were also reported to display such a thickening of the outer lipid membrane that may enable the pathogen to block antibiotics from entering inside the bacterial cell [46]. We postulate that, during dormancy, WE accumulate in the cell wall of *Mtb* to act as a permeability barrier that decreases nutrient uptake and limit growth. In support of this hypothesis, we found that deletion of *fcr1* and/or *fcr2* genes resulted in loss of WE synthesis and showed increased uptake of glycerol. We have previously shown that, under MS, *Mtb* accumulates WE and develops phenotypic antibiotic resistance [11]. Here we report that deletion of *fcr* genes causes *Mtb* cells to become incapable of accumulating WE and exhibit a nearly complete loss in the ability to develop phenotypic antibiotic resistance under MS. The loss of WE synthesis in the *fcr* deletion mutants probably leads to greater intake of nutrients leading to higher metabolic activity levels and growth rates in the mutants thereby resulting in the loss of phenotypic antibiotic tolerance.

To determine whether the higher nutrient uptake, metabolic activity and growth observed with the mutants subjected to MS, is reflected in the gene expression levels, we measured transcript levels of certain genes associated with catabolism, energy production and transcription. *GltA1* (citrate synthase I) and *citA* (citrate synthase II), that are known to be induced under stresses [11], [33], were more highly induced in Δ*fcr* mutants, compared to WT, under MS. This finding is consistent with the high level of active growth of the Δ*fcr* mutants compared to WT observed under MS condition. The upregulation of genes that are involved in energy generation (*nuoA*, *nuoB*, *nuoE*, *atpA*, *atpB*) and transcription (*rpoA*, *rpoB*) strongly suggest that the Δ*fcr* mutants are not able to shut down active growth when the ability to accumulate WE is compromised and thus these Δ*fcr* mutants are not able to develop the dormancy-related phenotypes under dormancy-inducing conditions *in vitro*.

Complementation of the Δ*fcr* mutants using pMV361 constructs with the respective native genes was only partially successful in restoring wax ester production in the *fcr* mutant cells under the stress conditions that promote WE production. Such partial restoration of lost *fcr* functions in the complemented mutants could not recover wax ester synthesis to WT levels under MS conditions. Consequently, the permeability barrier most likely remained defective in the mutants even after complementation and therefore [^14^C]glycerol (nutrient) uptake and growth remained high. Furthermore, the complemented mutants remained sensitive to antibiotics under MS.

Based on the results of this study and previous studies [6], [11], [12], [47], we conclude that TAG accumulation and WE accumulation that occur during stress conditions that mimic what the pathogen encounters within the host contribute to the development of dormancy. TAG accumulation may contribute to the development of a nonreplicating state due to the channeling of metabolites to TAG as recently suggested [48] whereas WE accumulation may inhibit replication by inhibiting nutrient uptake by the pathogen. It is likely that WE accumulation may be an important factor in the previously reported alterations of the *Mtb* outer lipid membrane during dormancy or in drug-resistant strains of *Mtb* [45], [46], [49]–[51]. Thus, the products of the *fcr* genes identified by the present work may indicate additional targets for candidate drugs that inhibit WE accumulation by the pathogen and thus prevent development of phenotypic tolerance to antimycobacterial drugs. Administration of drugs that inhibit WE synthesis together with currently used antibiotics could enhance elimination of *Mtb* and shorten the time of treatment required for curing tuberculosis.

## Supporting Information

Table S1 PCR primers used for *fcr* disruption in *Mtb*.
(DOC)
