# Supplementary material for: Wax Ester Synthesis is Required for Mycobacterium tuberculosis to Enter In Vitro Dormancy
Source: PLoS One. 2012 Dec 14;7(12):e51641. doi: 10.1371/journal.pone.0051641 (PMC3522743; doi:10.1371/journal.pone.0051641)
Supplement: Table S1 — PCR primers used for fcr disruption in Mtb. (DOC) [file pone.0051641.s001.doc]

**SUPPLEMENTARY INFORMATION**

**TABLE S1. PCR primers used for *fcr* disruption in *Mtb.***

|  |
| --- |
| Primer pairs to amplify 5’ and 3’-flanks of *fcr*1  5’-flank  *fcr*1A : 5’- CTTAAGGGTCTAGCCGTGCTCGCCGCG -3’  *fcr*1B: 5’- TCTAGACGATAAACCCGGTACCGCCGG -3’  3’-flank  *fcr*1C: 5’ - AAGCTTAGGATTTTGTGTCCAGACGC -3’  *fcr*1D: 5’- ACTAGTCTCGGATTGCAGCCGGGCATGA -3’ |
| Primer pair to amplify the deleted segment  Δ*fcr*1-F: 5’- GGTTCGCCGCCAGTCGTTAAGCCG -3’ Δ*fcr*1-R: 5’- GCGTAGGTGGCGAATTCGGGGACG -3 |
| Primer pairs to amplify genomic flanks in the mutants  5’-flank  GenA*fcr*1: 5’-CGCCGGACAGCAAATCACCGCA H1 : 5’-TGAGGCGATGGTGGTGTCGATGCT-3’ 3’-flank H2 : 5’-GGAACTGGCGCAGTTCCTCTGGGG-3’ GenD*fcr*1 : 5’-CGACGACATGACGCTGCAAGAG-3’ |
| **PCR primers used for *fcr*2 disruption in *M. tuberculosis* H37Rv** |
| 5’-flank  *fcr*2A 5’: CTTAAGGGTCGGTGTCTTGCGCACAAGC -3’  *fcr*2B 5’: TCTAGATGACGATGTTGGACACCGCCGC -3’  3’-flank  *fcr*2C: 5’- AAGCTTGCGGTGATGGACCGGGTACGTA -3’  *fcr*2D: 5 -ACTAGTCATCTGGCAGCACTGCGACTCG -3’ |
| Primer pair from the deleted segment  Δ*fcr*2-F: 5’- GGTGGGTCATCAGGCATCGGTGCG -3’ Δ*fcr*2-R: 5’-TACGTACCCGGTCCATCACCGCGG -3 |
| Primer pairs to amplify genomic flanks in the mutants  5’-flank  GenA*fcr*2: 5’-ACCAGCGGGTCTGGTAGGCCTCC-3’ H1 :-5’-TGAGGCGATGGTGGTGTCGATGCT-3’ 3’-flank  H2 : -5’-GGAACTGGCGCAGTTCCTCTGGGG-3’ GenD*fcr*2: 5’-AGTGACTTGTCGCGCTGCAGAGGGC-3’ |
